# Visualizing regulatory lymphocytic responses to predict neurological outcome after stroke

**DOI:** 10.1111/cns.13698

**Published:** 2021-06-22

**Authors:** Wanqing Xie, Peiying Li

**Affiliations:** ^1^ Department of Anesthesiology State Key Laboratory of Oncogenes and Related Genes Shanghai Cancer Institute Renji Hospital Shanghai Jiao Tong University School of Medicine Shanghai China

Acute ischemic stroke, one of the leading causes of death and disability all around world, induces profound activation of the lymphocytes which could interact with the ischemic brain injury and significantly impact stroke pathology. Meanwhile, stroke is common in elderly people who are at high risk of malignant cancer. Since the lymphocytic immune response participates both in tumor progression and brain injuries produced by ischemia, there might be a noteworthy reciprocal interaction between stroke and cancer. For example, the ischemic brain could induce an immunosuppressive response that may promote cancer progression and trigger fetal infections which seriously affect the prognosis and survival of stroke patients. Therefore, we need more precise indicators to maintain the balance of the post‐stroke immunity and predict the outcome of cancer‐bearing stroke patients receiving immunotherapies.

Lymphocytes play an important role in the pathological mechanism of adaptive immune response after stroke. Within hours of transient middle cerebral artery occlusion (tMCAO), T lymphocytes infiltrate into the brain and gather near the boundary of the infarction area. In particular, CD4^+^ T cells infiltrate into the ischemic brain within 24 h and peaked at 72 h after reperfusion, which induce brain injury and exacerbate neuroinflammation.^1^ Cytotoxic CD8^+^ T cells can be recruited into the brain as early as 3 h after stroke and can exacerbate the neurological deficits up to weeks after ischemic stroke by inducing demyelination of the neuronal axons.^2^ To avoid overactivation of the neuroinflammation, the body has self‐limiting mechanism to counteract the T‐cell‐mediated immune response in the brain. The activation of the immune regulatory cells, such as regulatory T cells (Tregs) and regulatory B cells (Bregs), represents one of the critical self‐limiting mechanism.^3^, ^4^ Regulatory B cell is a subset of B lymphocytes with immunomodulation function, which plays an important role in maintaining immune tolerance and suppressing harmful immune response. Bregs can limit CNS inflammation and neurologic deficits in murine experimental stroke.^4^ CD4^+^Tregs account for only 5% of the CD4^+^ T cells and are associated with a variety of inflammatory response pathways and have neuroprotection effects on inflammatory responses after ischemic stroke, and IL‐10 signaling is essential in the immunomodulation of Treg cells.^5^ Hence, Treg cells are significant modulators of inflammatory‐induced brain injury after ischemic stroke. CD8^+^ Treg is a small subset of T cells that is obtaining increasing attention in recent years. It has been shown to play important roles in controlling several autoimmune diseases, such as CD4^+^‐cell‐induced colitis, experimental autoimmune encephalomyelitis, experimental inflammatory bowel disease, and autoimmune type 1 diabetes. However, the role of CD8^+^ Tregs in ischemic brain injury has not been reported.

In the paper by Li et al.,^6^ the percentage of circulating T cells, CD4^+^ T cells, CD8^+^ T cells, double‐negative T cells (DNTs), CD4^+^Tregs, CD8^+^Tregs, B cells, and Bregs in the peripheral blood were examined both at admission and 3 months after stroke by enrolling a total of 210 acute ischemic stroke patients and 876 healthy controls. The researchers found that the number of B cells, Bregs, and CD8^+^Tregs increased significantly, while CD4^+^ Tregs dropped and soon reversed after ischemic stroke. Notably, CD4^+^Tregs, CD8^+^ Tregs, and DNTs exhibited high correlation with the infarct volume and neurological scores of stroke patients. The percentage of CD4^+^ Tregs within lymphocytes displayed high correlations with both acute and long‐term neurological outcomes, which exhibited a great independent predictive ability. Our previous study reported that adoptive transfer of CD4^+^Tregs can protect the integrity of the blood‐brain barrier and thus attenuates the ischemic brain injury and reduces the risk of tPA‐induced hemorrhagic transformation after stroke.^7^, ^8^ The participation of adaptive immunity in the pathological process of ischemia and reperfusion and the protective effect of regulatory lymphocytes in stroke is proved by many researchers. There is massive accumulation of Treg cells in the mouse brain after ischemic stroke, and this potentiates neurological recovery during the chronic phase of ischemic brain injury.^3^ Stroke induced significant bilateral B‐cell diapedesis into remote brain regions and mediated motor and cognitive recovery^9^ Although much has been learned on the interaction between immune responses and damaged brain and factors affecting neurological outcome, it is still unclear how the regulatory lymphocytic responses develop in the brain in the short term and long term after stroke. In addition, biomarkers which could be applied directly in clinic still remain deficient. Identifying specific biomarkers of the lymphocytic responses after stroke could guide the clinical management, attenuate long‐term neurological deficit, and improve long‐term life quality of stroke patients.

All the above evidence strongly supports CD4^+^ Tregs could serve as a sensitive biomarker and also a potential therapeutic target for stroke therapy. However, as an immune suppressive T‐cell subset, CD4^+^Tregs could exacerbate cancer progression, which raises a concern for the translation of Treg‐based stroke therapy, especially in cancer‐bearing stroke patients. Recent evidence suggests that in cancer‐bearing stroke mice, CD4^+^ Tregs could be recruited into the tumor, thus attenuate their protection of the blood‐brain barrier after stroke.^10^ Importantly, with the aging of the population, the number of cancer patients is continuously rising. Therefore, cancer‐related immune response is emerging as a noteworthy clinical issue for stroke patients. Considering the importance of the regulatory lymphocytes in the severity of ischemic stroke, developing blood‐based biomarkers targeting above mentioned regulatory immune cells could provide valuable and easily accessible predictive tool to predict stroke outcome and improve stroke patient management.

## CONFLICT OF INTEREST

The author(s) declared no potential conflicts of interest with respect to the authorship and/or publication of this article.

## AUTHOR CONTRIBUTIONS

WX drafted the manuscript. PL revised the manuscript.

## Data Availability

All data are available upon request by contact with the corresponding author.
